# Blood Transfusion

**Published:** 1919-08-02

**Authors:** 


### BLOOD TRANSFUSION.

### An Outline of the Technique and Applications.

Now that so many medical men have seen the use and advantages of whole-blood transfusion as recently applied in the war areas, there is no doubt that the procedure will be considered as having far more general utility in civil work. Great strides have already been made in the American hospitals, and the results there obtained, even without any of the confirmatory evidence provided by our own experience, establish the more extensive employment of this method as one of the most definite medical advances made during the past eighteen months.

### Severe Primary Hæmorrhage.

As might be expected, the most spectacular successes have been obtained when the patient has suffered considerable loss of blood. Transfusion then finds its most obvious applications, but, although much of our experience has been gained from the traumatic injuries of war, a moment's reflection will bring to mind the enormous number of clinical conditions in which extensive bleeding, internal or external, may have occurred, and likely, therefore, to benefit greatly from judicious reinforcement of the circulating blood. There have been a number of controversies on the relative merits of saline, gum-solutions, and whole-blood, and, at first sight, the arguments employed are a little confusing to the reader. The point is, however, largely a matter of circumstance, and, we believe, the majority of the profession will agree with our views on the subject. With saline and gum arabic, as applied to shock or hæmorrhage, or both, their chief claim to value is ease of preparation and the possibility of using them in difficult surroundings and outside hospital practice; but both are definitely inferior to whole-blood, transfusion of which should be preferred wherever circumstance and organisation permit. There is support for this statement in both clinical and animal experimental experience, and the relative merits of saline and gum themselves need not concern us here. According to Makins:

> While it may be stated that blood is the best fluid with which to replace lost blood, yet in practice this may be both impossible and unnecessary. With a moderate hæmorrhage there is no need to replace the lost blood artificially. If the bleeding has been severe the loss can be made good by a more easily obtainable fluid, *i.e.*, Bayliss's 6-per-cent. gum-arabic solution. A still more severe hæmorrhage will demand blood.

Unfortunately we still have no precise indication as to when the use of blood becomes imperative. A critical point is probably reached when the total hæmoglobin content falls as low as 30 per cent., but this determination is difficult in an emergency. In any case, it is hard to tell from a patient's appearance how much actual loss has occurred, and it is suggested that, under uncertain circumstances, the blood pressure should be taken. A systolic pressure remaining below 80 m.m. of mercury is strong indication immediately to provide assistance to the circulatory system. In settling the question of what nature this aid should be, one may summarise by saying that (*a*) in abundant and sudden bleeding, immediate transfusion of blood is needed; (*b*) in less severe cases, gum-solution may be tried, and if no permanent good results from it then follow up with transfusion; (*c*) the mildest instances should be given fluids by mouth and rectum whenever possible, holding method (*b*) in reserve.

### Secondary Hæmorrhage.

In the common circumstances of secondary hæmorrhage, the hæmoglobin content is usually already so depressed that even a relatively slight hæmorrhage will reduce it to a degree sufficient seriously to prejudice recovery, and most workers will agree that blood transfusion is then the best method. In consulting published records it must be remembered that if the patient's infection be generalised a spectacular recovery must not be expected. But here we come to a most important application of transfusion. In the case of the generalised infections it is frequently possible to obtain in pure culture the causal organism of the septicæmia, the healthy donor can often be immunised to a high degree against these organisms, and, this accomplished, the transfusion may be made with marked benefit to the patient, quite apart from any hæmorrhage occurring previously. A number of excellent results from such procedure have been published, and if one reflects on the great number of septicæmias which occur in civil practice the long, chronic, and often fatal, course which many of them run, thus giving time and excuse for the somewhat lengthy technique to be completed, it is at least a suggestion of possible utility that our hospitals should attempt more frequently to apply this method.

### Anæmias and Other Conditions.

Taking a disease with such small percentage recovery as pernicious anæmia, it is noteworthy that both Unger and Kimpton report on series of cases in which a quarter of the number of sufferers were apparently cured and several received considerable benefit. They suggest that the result is produced not so much by a direct increase in the oxygen-carrying power or volume of the blood as by healthy stimulation to renewed activity of the red marrow with a resulting remission in the anæmia. For the ordinary secondary anæmias great benefit has resulted, and a most satisfactory reduction in periods of convalescence, etc., is produced, but, as might be expected from the pathology, in the leukæmic and lymphatic types no success has been obtained.

### Essentials of Technique.

(*a*) *Direct transfusion* is the original, and probably the worst, means of getting the blood into the veins of a sick man. Apart from difficulties of manipulation, it is very hard to tell exactly how

**Blood Transfusion**—(*continued*).

much blood has passed, and often, owing to venous spasm, it is doubtful whether any transfusion at all has occurred. Determination of the length of time required is extremely difficult, and in view of improvements described later there is general agreement that the direct method should be abandoned.

(*b*) *Transfusion of Unmodified Blood.*—Indirect transference is then to be preferred, but comparison is still to be made between citrated and unmodified blood. There is still some difference of opinion as to which is the safer and more physiological, but one has seen both methods most successfully employed, and our only comment is that with unmodified blood there is certainly greater risk of massive clotting in the glass receptacle and failure of the operation, in spite of the donor's sacrifice, and also that it is essential in this method that donor and recipient shall be brought together, which is not invariably desirable. Most of the present technique is modified from Kimpton's original, as briefly outlined below:

A glass cylinder of about 500 c.c. is drawn out to a cannula-point below. To the top is attached a small hand-pump (rubber), which, when the cannula is inserted in the donor's vein, sucks up the blood, and, when in the receiver's vein, drives the blood out again at any required speed. The cylinder is sterilised and paraffin filmed by boiling in water with a layer of paraffin on the surface; the cylinder is removed by being drawn out of the water in a vertical position, so that a perfect paraffin film adheres to its inner surface. In contact with an unbroken film the blood remains unclotted for ten to fifteen minutes.

(*c*) *Transfusion of Citrated Blood.*—Rendle Short described a neat and efficient citrate method, comprising a bottle holding 20 to 30 ounces, partially paraffined inside by inserting a small piece of wax, m.p. 42°C, before autoclaving, and into which is run 160 c.c. of isotonic sodium citrate solution. The bottle functions in the same way as the Kimpton's tube, but in this instance the transfusion may, provided all tubes and apparatus be scrupulously clean, be performed without cutting down on the veins, a needle being used for both donor and receiver. This is, to our mind, a considerable advantage, as also the fact that the citrated blood keeps safely for half an hour or more and can be transferred some distance to the patient.

(*c*) *Preserved Red Cells.*—Although it is impossible to keep blood plasma any length of time without it undergoing toxic changes, Rous and Turner have shown that red cells will keep in a citrate-dextrose solution for several weeks, and elaboration of their discovery received considerable and successful application in the latter part of the war. The life-saving effect was apparently as good as with whole-blood methods, but essentially this is a war procedure and will probably find but small application in civil work.

### Dangers of the Method.

Risks incurred fall almost entirely upon the receiver. For example, incompatibility of blood is important. If they are not tested there is about a 30-per-cent. risk of hæmolysis, and in such cases 5 to 10 per cent. may show alarming or fatal reaction. Syphilis may be conveyed, but the danger should be excluded by taking a Wassermann. Also malaria might be transmitted, but care in history investigation should obviate this danger. Even with tested bloods there is in the experience of Kimpton, and others, a very small percentage risk of marked hæmolysis and reaction, but with modern technique even this is decreasing, and otherwise in normal cases the chief signs are a short initial pyrexia, a symptomless polymorphoneuclear leucocytosis, and an urticarial rash.

### Compatibility of Bloods.

The blood of any individual has the characters of one of four groups, and these apparently persist unchanged throughout life, but consanguinity does *not* guarantee that the bloods fall into the same group from the transfusion point of view. The blood of a person belonging to a certain group may be given with safety to another in the same group, but not necessarily to one in another. The reaction lies in both agglutination and hæmolysis, and it is established that a blood which will hæmolyse another will also agglutinate it, and hence, for convenience, use is made of the latter phenomenon.

An approximate occurrence of the types is:—

| | Suitable for Patient of |
|---|---|
| Group I.—5 per cent. | Group I. |
| Group II.—40 per cent. | Group I, II. |
| Group III.—10 per cent. | Group I, III. |
| Group IV.—45 per cent. | Group I., II., III., IV. |

What matters is the effect of the receiver's plasma on the donor's cells, not the reverse, this indifference being probably due to the great bulk of the receiver's blood in comparison with the amount transfused. In choosing donors, therefore, use is made either of one belonging to the same group as the patient or else one in Group IV. Group IV. are seen above to be universal providers, and may be used with safety for any case, otherwise testing must be done.

Stock Group II. and Group III. sera may be kept, and by macroscopic drop methods agglutination observed. To the donor's cells are added the known sera, and the results prove in the following way:—

Donor's cells agglutinated by serum II. and serum III., then donor is Group I.
Donor's cells agglutinated by serum III., *not* by serum II., then donor is Group II.
Donor's cells agglutinated by serum II., *not* by serum III., then donor is Group III.
Donor's cells are not agglutinated by either, then donor is Group IV.

This scheme gives an absolute classification for the donor, but if, as must often occur, stock sera are not at hand and the surgeon must determine the suitability of a would-be donor, a very simple procedure may be adopted. A few c.c. of blood is drawn from the patient, the clear serum separated and a little citrate added. With a drop of this on a glass slide mix in a *very little* of the donor's blood. If there is agglutination in five minutes the donor is unsuitable, if there is no agglutination the donor's blood may be used for the transfusion of the patient concerned.

